# Follow-up analysis of quality of life in elderly patients with bone trauma: a longitudinal observational study

**DOI:** 10.1186/s12877-023-04325-y

**Published:** 2023-09-27

**Authors:** Xiaofeng Xu, Qixiang Zheng, Siying Wei, Yongmei Chen, Xiuying Hu

**Affiliations:** 1 Division of Vascular Surgery, Department of General Surgery, Innovation Center of Nursing Research and Nursing Key Laboratory of Sichuan Province, West China Hospital, Sichuan University/West China School of Nursing, Sichuan University, Chengdu, 610041 China; 2 Division of Vascular Surgery, Department of General Surgery, West China Hospital, Sichuan University/West China School of Nursing, Sichuan University, Chengdu, 610041 China; 3 West China School of Nursing, Sichuan University, Chengdu, 610041 China; 4 Division of Vascular Surgery, Department of General Surgery, West China Hospital, Sichuan University, Chengdu, 610041 China; 5 Innovation Center of Nursing Research and Nursing Key Laboratory of Sichuan Province, West China Hospital, Sichuan University/West China School of Nursing, Sichuan University, Chengdu, 610041 China

**Keywords:** Elderly, Bone trauma, Quality of life, SF-36

## Abstract

**Background:**

The quality of life (QoL) of elderly patients with bone trauma is significantly decreased and is affected by many complex factors. This study aims to conduct a half-year follow-up survey to clarify QoL and its influencing factors in elderly patients with bone trauma in order to provide targeted care measures for elderly patients with bone trauma.

**Methods:**

This was a longitudinal observational study. We used the 36-Item Short Form Health Survey (SF-36) to investigate and evaluate the QoL of 100 patients with bone trauma at the time of hospital discharge and 1 month, 3 months, and 6 months after discharge. Our previous study confirmed that the SF-36 had higher reliability and validity for evaluating the QoL of elderly patients with bone trauma. At the same time, we also investigated the age, gender, location of bone trauma, and destination after discharge of those patients. Those factors that might affect the QoL of elderly patients with bone trauma were identified by univariate and multivariate analyses.

**Results:**

The total physiological function, role-physical, bodily pain, vitality, social functioning, role-emotional, and mental health scores of elderly patients with bone trauma gradually increased from the time of discharge to 1 month, 3 months, and 6 months after discharge, and there were significant differences (*p* < 0.001). However, there was no significant difference in the general health score in the different periods (*P* = 0.095). The total QoL scores also significantly differed (*F* = 118.61, *P* < 0.001) at the time of discharge (335.252 ± 127.572) and 1 month (285.149 ± 112.827), 3 months (479.344 ± 153.663), and 6 months after discharge (544.396 ± 166.536). The univariate analysis results showed that the location of bone trauma (*P* < 0.005) and the destination after discharge (*P* < 0.001) were the main factors affecting QoL in different periods. The results of the multivariate analysis showed that the location of bone trauma was an important factor affecting QoL (*P* < 0.005 in different periods). Whether to undergo surgery was a factor affecting the patients’ long-term QoL (*P* < 0.005 at 6 months after discharge).

**Conclusions:**

Although the QoL of elderly patients with bone trauma gradually improves after injury, their recovery time is long, and the influencing factors are complex. Follow-up services should continue for at least six months for these patients, and comprehensive treatment and long-term rehabilitation services should be provided.

## Background

With the rapid development of related industries such as manufacturing and transportation, the occurrence of industrial accidents, traffic accidents, natural disasters, etc., has increased, and the number of patients with bone trauma has also increased significantly [1, 2]. Bone trauma can be divided into different types according to the injury site, including limb, clavicle, and lumbar fractures [3]. Elderly people have decreased bone density, mineral loss, and decreased muscle protection, so they are more prone to fractures; due to improvements in the treatment of chronic diseases, an increasing number of elderly people have a more active lifestyle, making the elderly population more vulnerable to injury [4–6]. Coupled with increased life expectancy, bone trauma is causing more elderly people to present to emergency departments [7]. It has been found that more than 50% of elderly trauma patients have single or multiple fractures [8].

Elderly patients with bone trauma often experience pain, swelling, limb deformities, dysfunction, abnormal daily activities, etc., which seriously affect their physical and mental health. Bone trauma causes malnutrition, dysfunction, and even disability, which seriously reduce quality of life (QoL) [9]. QoL is affected by many factors. A three-year survey of 5057 elderly patients with low-trauma fractures in Canada showed that despite a rebound in health-related quality of life (HRQoL) one month after the fracture, HRQoL will permanently decrease in the long term. Mobility had the greatest impact on HRQoL changes [10].

However, at present, China lacks specific tools to evaluate the QoL of elderly patients with bone trauma, and there is also a lack of in-depth and long-term follow-up research on the QoL of elderly patients with bone trauma. At the same time, there is a lack of comprehensive research on the factors influencing QoL in elderly patients with bone trauma. Thus, the QoL of elderly patients after bone trauma and its influencing factors in China are worth exploring and analyzing. In this research, we intended to analyze the QoL of elderly patients with bone trauma and its influencing factors through a six-month follow-up survey in a large general hospital in western China. This study could provide a reference for improving the QoL of elderly patients with bone trauma.

## Methods

### Study design and sampling

This study was a longitudinal observational study and obtained ethical approval. We adopted a convenient sampling method and selected elderly patients with bone trauma who were hospitalized in the trauma medical center of a comprehensive tertiary hospital in Chengdu between November 2021 and June 2022. The inclusion criteria were as follows: patients aged ≥ 60 years old who were admitted to the hospital due to bone trauma, conscious and expressive patients who had never used anti-anxiety or anti-depressant drugs, and patients who were informed and willing to cooperate with the investigator. The exclusion criteria were patients with mental symptoms and communication difficulties and discharged patients who were neglected by family members. The number of samples should be at least 5–10 times the number of variables. Through the previous literature review, approximately 10 related factors affecting QoL were identified. Considering a loss rate of 10%-15%, the sample size should be 58–118 people. Finally, the sample size of the follow-up survey was determined to be 100 elderly patients with bone trauma. The average age of the participants was 72.30 ± 9.702 years, and the average length of hospitalization was 10.82 ± 8.278 days. Five of the 100 patients died on the 4th, 6th, 14th, 107th and 184th days after injury.

### Survey tool

At present, there is no specific survey tool for evaluating the QoL of elderly patients with bone trauma. Our research team reviewed the literature and used the 36-Item Short Form Health Survey (SF-36), the 12-Item Short Form Health Survey (SF-12) and the EuroQol Five Dimensions Questionnaire (EQ-5D) to evaluate and compare the QoL of 157 elderly patients with bone trauma. The Cronbach coefficients of the SF-36, SF-12 and EQ-5D were 0.877, 0.701, and 0.393, respectively, and the construct validity (the degree to which a test measures the theoretical structure and traits to be measured) Kaiser‒Meyer‒Olkin (KMO) values were 0.762, 0.697, and 0.612, respectively. Therefore, the SF-36 had higher reliability and validity in evaluating elderly patients with bone trauma and was used as the follow-up survey tool for this research [11]. This tool includes the following 8 dimensions, which belong to two categories (physical health and mental health): physiological function (PF), role-physical (RP), bodily pain (BP), general health (GH), vitality (VT), social functioning (SF), role-emotional (RE) and mental health (MH). The higher the SF-36 score is, the better a patient’s physical function and mental state [12, 13].

At the same time, we also collected basic information about the patients, such as age, gender, location of bone trauma, education level, monthly income, whether they underwent surgery, and destination after discharge. Therefore, we can analyze the factors that affect the QoL of patients. These data were obtained by surveying patients during their admission period.

### Data collection

The QoL of the included elderly bone trauma patients was investigated and evaluated at the time of discharge and 1 month, 3 months, and 6 months after discharge. The survey content included the general information of the patients (age, gender, surgical status, education level, occupation, etc.) and their SF-36 scores. The investigators were clinical nurses who had been uniformly trained. The training included an explanation of the SF-36 scale, how to use SF-36 to evaluate elderly bone trauma patients, telephone follow-up methods, and communication skills between nurses and patients. The QoL assessment was completed in person when the patients were discharged from the hospital, and their QoL was assessed by telephone follow-up at 1, 3, and 6 months after discharge.

### Statistical analysis

After the follow-up data were collected and sorted, SPSS 26.0 statistical software was used for analysis and processing. The QoL score of the patient was expressed as the mean ± standard deviation. The patients’ QoL scores in different periods were compared by one-way repeated measures analysis of variance. Univariate and multivariate analyses were conducted for the relevant factors influencing QoL in different periods. The log rank method was adopted for univariate analysis, and multiple linear regression analysis was adopted for multivariate analysis. *P* < 0.05 indicated that the difference was statistically significant.

## Results

### QoL scores of the patients in different periods

**Fig. 1:**
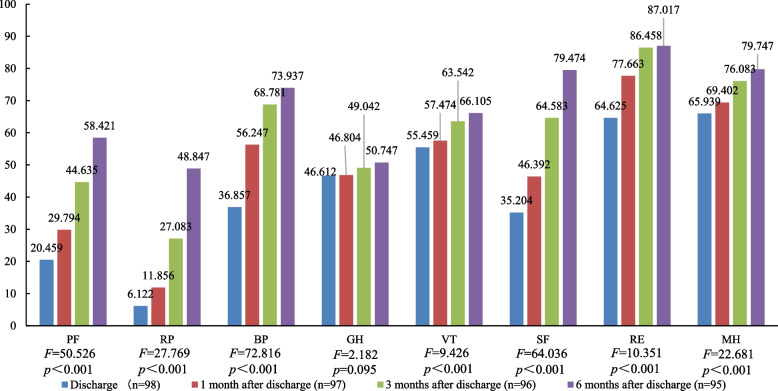
QoL scores of elderly patients with bone trauma in different periods

For the 8 dimensions included in the SF-36, the scores of physiological function, role-physical, bodily pain, vitality, social functioning, role-emotional and mental health were significantly different at the time of discharge and 1 month, 3 months, and 6 months after discharge (*P* < 0.001) (Fig. 1). However, there was no significant difference in general health score among the different periods (*P* = 0.095) (Fig. 1). The total QoL scores of the SF-36 were also significantly different (*F* = 118.61, *P* < 0.001) at the time of discharge (335.252 ± 127.572) and 1 month (285.149 ± 112.827), 3 months (479.344 ± 153.663), and 6 months after discharge (544.396 ± 166.536). In addition, the patients’ QoL scores decreased 1 month after discharge and then gradually increased.

### Analysis of factors influencing patients' QoL

Log rank test results for univariate analysis showed that the patients’ QoL was mainly related to the location of bone trauma and the destination after discharge (Table 1).

**Table 1:** Univariate analysis of influencing factors on QoL in different periods (*n* = 100)

Project	Category	Number of patients	Discharge		1 month after discharge		3 months after discharge		6 months after discharge	
			QoL score	*F/p*	QoL score	*F/p*	QoL score	*F/p*	QoL score	*F/p*
Age (years)	60–80	77	329.515 ± 15.488	0.221/0.638	283.864 ± 13.288	0.044/0.834	473.314 ± 17.351	0.740/0.390	533.595 ± 19.349	2.855/0.091
	> 80	23	337.150 ± 21.810		292.234 ± 20.720		503.763 ± 36.020		590.602 ± 34.716	
Gender	Male	35	330.242 ± 24.510	0.024/0.877	297.751 ± 16.642	0.036/0.850	515.005 ± 24.687	2.585/0.108	568.279 ± 28.809	1.845/0.174
	Female	65	331.612 ± 15.270		279.443 ± 14.924		461.508 ± 19.632		532.828 ± 21.181	
Location of bone trauma	Upper limbs	30	387.594 ± 25.570	36.783/ < 0.001	317.983 ± 21.191	19.153/0.001	529.277 ± 29.169	9.993/0.041	585.744 ± 28.933	10.092/0.039
	Lower limbs	51	326.100 ± 16.526		281.468 ± 14.660		468.866 ± 21.299		550.304 ± 22.399	
	Pelvis	2	129.165 ± 17.165		152.000 ± 18.00		371.085 ± 98.915		348.000 ± 198.000	
	Compound injury	15	262.577 ± 26.756		232.911 ± 23.793		421.533 ± 32.243		466.821 ± 47.970	
	Clavicle	2	289.080 ± 76.750		355.000 ± 204.000		420.665 ± 232.235		425.415 ± 171.128	
Surgical status	Had surgery	80	338.202 ± 14.938	2.315/0.128	294.337 ± 11.541	0.382/0.536	492.711 ± 16.520	2.008/0.157	558.500 ± 18.430	3.945/0.047
	Did not have surgery	20	301.833 ± 24.738		247.306 ± 33.550		421.398 ± 40.665		484.064 ± 41.948	
Chronic disease	None	35	340.547 ± 25.624	4.914/0.178	302.062 ± 20.283	5.974/0.113	481.219 ± 25.891	1.630/0.653	565.779 ± 27.130	2.691/0.442
	One	39	332.923 ± 18.711		258.291 ± 15.437		483.354 ± 25.270		533.273 ± 28.858	
	Two	17	335.468 ± 30.180		335.666 ± 24.978		519.122 ± 22.472		561.022 ± 29.958	
	Three or more	9	272.770 ± 31.306		241.143 ± 51.790		362.333 ± 77.010		466.880 ± 83.372	
Body Mass Index (BMI)	< 18.5	9	342.747 ± 25.937	4.324/0.229	246.444 ± 23.177	4.712/0.194	459.851 ± 54.938	3.819/0.282	527.629 ± 58.352	1.696/0.638
	18.5–23.90	49	304.407 ± 18.333		285.174 ± 17.200		463.540 ± 20.724		541.129 ± 26.017	
	24.0–27.90	31	360.978 ± 25.839		304.392 ± 22.087		498.070 ± 31.194		538.414 ± 31.017	
	≥ 28.0	11	351.955 ± 35.843		285.545 ± 24.144		507.136 ± 43.993		588.045 ± 32.610	
Ethnicity	Han	97	333.737 ± 13.058	3.306/0.069	287.812 ± 11.471	5.495/0.019	482.603 ± 15.610	2.540/0.111	545.545 ± 17.416	1.934/0.164
	nationality	3	207.000 ± 95.000		181.000 ± 11.000		326.000 ± 144.000		491.000 ± 55.000	
Education level	Primary school and less	43	306.971 ± 18.141	6.305/0.098	265.634 ± 16.400	5.927/0.115	485.024 ± 21.080	2.359/0.501	529.275 ± 27.263	4.226/0.238
	Junior high school	21	307.317 ± 26.585		291.929 ± 29.703		434.291 ± 41.610		492.258 ± 42.514	
	High school/technical secondary school	16	362.678 ± 37.146		265.813 ± 19.506		474.844 ± 35.154		577.365 ± 32.994	
	Undergraduate/associate’s degree	20	384.394 ± 29.790		338.404 ± 25.259		518.281 ± 36.883		603.351 ± 32.105	
Marital status	Unmarried	1	334.000 ± 0.000	7.882/0.049	294.000 ± 0.000	6.045/0.109	583.000 ± 0.000	0.437/0.933	554.000 ± 0.000	1.153/0.764
	Married	74	337.671 ± 15.839		297.097 ± 13.658		474.729 ± 18.016		535.815 ± 19.994	
	Divorce	1	169.830 ± 0.000		245.000 ± 0.000		462.000 ± 0.000		584.000 ± 0.000	
	Widow	24	316.424 ± 21.590		246.667 ± 18.678		492.083 ± 34.424		573.258 ± 35.623	
Way of living	Living with spouse and children	27	313.166 ± 24.464	2.662/0.616	300.395 ± 23.971	1.445/0.836	463.907 ± 28.428	5.898/0.207	533.457 ± 30.357	7.469/0.113
	Living with spouse	38	346.403 ± 23.220		285.053 ± 17.440		483.732 ± 26.844		535.788 ± 31.153	
	Living with children	18	340.958 ± 24.767		272.200 ± 21.292		554.143 ± 34.811		646.512 ± 25.994	
	Live alone	9	306.926 ± 32.505		281.500 ± 44.560		421.037 ± 42.356		498.722 ± 50.860	
	Others	8	327.041 ± 56.456		268.125 ± 45.744		445.250 ± 54.115		493.813 ± 54.604	
Monthly income (CNY)	< 1000	27	328.000 ± 23.879	2.454/0.653	293.167 ± 15.563	0.257/0.992	534.373 ± 28.240	4.981/0.289	608.646 ± 28.863	7.368/0.118
	1000–3999	35	338.057 ± 22.237		284.562 ± 21.618		471.395 ± 24.834		524.057 ± 28.676	
	4000–6999	27	327.820 ± 26.551		278.545 ± 23.697		449.058 ± 33.569		535.160 ± 34.981	
	7000–9999	6	296.900 ± 21.729		295.600 ± 16.717		447.500 ± 29.607		504.266 ± 42.044	
	≥ 10,000	5	351.432 ± 80.919		280.400 ± 74.158		449.100 ± 85.380		466.532 ± 95.836	
Payment method of treatment cost	Medical insurance + partial self-expense	78	344.300 ± 14.561	7.036/0.071	297.095 ± 12.669	6.374/0.095	499.022 ± 16.902	15.729/0.001	565.317 ± 18.518	13.911/0.003
	Commercial medical insurance	1	196.830 ± 0.000		167.000 ± 0.000		195.830 ± 0.000		267.330 ± 0.000	
	Self-expense	10	252.533 ± 43.414		248.800 ± 27.878		431.282 ± 52.273		473.832 ± 64.480	
	Others	11	323.985 ± 34.732		251.545 ± 41.192		416.340 ± 15.600		486.396 ± 49.042	
Living area	Town	71	346.490 ± 15.954	5.102/0.024	293.993 ± 14.444	2.437/0.118	468.872 ± 20.098	0.004/0.951	549.223 ± 19.524	0.207/0.649
	Country	29	292.803 ± 20.493		264.952 ± 16.345		504.762 ± 21.633		532.240 ± 35.096	
Occupation	Worker	2	279.250 ± 72.420	5.959/0.545	208.500 ± 95.500	8.293/0.308	390.335 ± 58.665	30.551/ < 0.001	384.165 ± 202.835	6.799/0.450
	Famer	22	334.424 ± 29.444		288.970 ± 23.346		522.530 ± 27.103		546.757 ± 40.397	
	Professional technician	2	244.165 ± 74.335		247.500 ± 2.500		416.835 ± 45.165		588.500 ± 4.500	
	Civil servant	1	302.000 ± 0.000		192.000 ± 0.000		182.000 ± 0.000		436.000 ± 0.000	
	Retired personnel	51	343.048 ± 18.802		298.813 ± 16.829		477.898 ± 23.164		550.932 ± 22.841	
	Unemployed	4	390.500 ± 42.442		317.667 ± 52.235		548.167 ± 48.499		631.333 ± 39.253	
	Freelance	3	337.223 ± 33.179		304.667 ± 69.671		611.000 ± 43.509		664.333 ± 39.253	
	Others	15	290.571 ± 33.228		245.369 ± 26.605		416.440 ± 41.066		494.231 ± 48.176	
Destination after discharge	Transfer to subordinate hospital	24	250.499 ± 21.366	20.451/ < 0.001	224.153 ± 23.818	6.919/0.031	374.293 ± 29.728	18.829/ < 0.001	423.750 ± 34.524	18.665/ < 0.001
	Home	71	363.134 ± 14.469		306.920 ± 12.120		516.328 ± 16.274		585.178 ± 17.266	
	Death	5	203.915 ± 105.915		309.000 ± 0.000		/		/	

Multivariate analysis showed that the main factors affecting the patients’ QoL at discharge were the location of bone trauma and the destination after discharge. The main factors influencing QoL at 1 month after discharge were marital status and destination after discharge. The main factors influencing QoL at 3 months after discharge were sex and destination after discharge, and the main factors influencing QoL at 6 months after discharge were surgical status, age, education level, monthly income, and destination after discharge (Table 2).

**Table 2:** Multivariate analysis of factors related to QoL in elderly patients with bone trauma in different periods (*n* = 100)

Variable	Discharge				1 month after discharge				3 months after discharge				6 months after discharge			
	*B*	*SE*	*t*	*p*	*B*	*SE*	*t*	*p*	*B*	*SE*	*t*	*p*	*B*	*SE*	*t*	*p*
Surgical status	-23.083	36.644	-0.630	0.531	-27.719	31.609	-0.877	0.383	-63.693	39.87	-1.597	0.114	-86.616	40.567	-2.135	0.036
Hospitalization days	-0.180	1.819	-0.099	0.921	-0.703	1.566	-0.449	0.655	-2.431	1.982	-1.227	0.224	-2.197	2.016	-1.09	0.279
Gender	-19.762	29.655	-0.666	0.507	-28.415	25.507	-1.114	0.269	-77.358	32.559	-2.376	0.020	-53.126	33.142	-1.603	0.113
Age	0.446	1.558	0.286	0.776	1.229	1.353	0.909	0.366	1.905	1.721	1.107	0.272	3.898	1.766	2.208	0.03
Chronic disease	-15.183	15.288	-0.993	0.324	-5.439	13.233	-0.411	0.682	-21.762	16.683	-1.304	0.196	-33.519	17.12	-1.958	0.054
Location of bone trauma	-27.261	13.481	-2.022	0.047	-12.234	11.627	-1.052	0.296	-15.599	14.670	-1.063	0.291	-12.548	14.978	-0.838	0.405
Ethnicity	-64.516	95.252	-0.677	0.5	-55.135	81.996	-0.672	0.503	-59.819	103.892	-0.576	0.566	144.415	105.941	1.363	0.177
Education level	16.840	15.703	1.072	0.287	14.212	13.592	1.046	0.299	20.952	17.153	1.221	0.226	56.717	17.46	3.248	0.002
Marital status	-20.742	20.517	-1.011	0.287	-40.489	17.693	-2.288	0.025	-4.608	22.491	-0.205	0.838	26.087	22.923	1.138	0.259
Way of living	8.119	14.141	0.574	0.567	5.595	12.171	0.460	0.647	5.945	15.358	0.387	0.700	-19.057	15.615	-1.12	0.226
Monthly income	-15.791	16.067	-0.983	0.329	-16.37	14.076	-1.163	0.248	-23.913	17.876	-1.338	0.185	-53.692	18.349	-2.926	0.005
Payment method of treatment cost	-3.114	13.957	-0.983	0.329	-5.749	12.005	-0.479	0.633	-8.444	15.153	-0.557	0.579	12.143	15.507	0.783	0.436
Residence	-29.287	38.057	-0.770	0.444	-8.186	32.739	-0.25	0.803	62.908	41.65	1.510	0.135	22.73	42.856	0.53	0.597
Occupation	-0.702	4.688	-0.150	0.881	-0.42	4.032	-0.104	0.917	1.085	5.077	0.214	0.831	3.138	5.257	0.597	0.552
Destination after discharge	63.511	30.483	2.083	0.040	64.828	27.077	2.394	0.019	126.708	35.669	3.552	0.001	159.495	36.409	4.381	< 0.001
BMI	10.289	16.993	0.606	0.547	1.722	14.618	0.118	0.907	19.563	18.402	1.063	0.291	9.197	18.713	0.491	0.624

## Discussion

Elderly patients are more likely to be injured due to minor accidents than younger people, and they experience greater difficulty compensating for injuries caused by trauma [14]. Because of multimorbidity, elderly patients are more likely to use multiple drugs to treat chronic diseases, some of which may reduce their response to traumatic physiological stress, thereby increasing the risk of complications [15]. As a result, older trauma patients also have a higher risk of severe disability and death [16]. The incidence of bone trauma is high in elderly patients.

The results of this study showed that among the eight dimensions of the SF-36, only the general health score showed no difference, while the scores of the other seven dimensions gradually increased. The mental health and role-emotional scores were higher, while the role-physical and physiological function scores were lower. The results are consistent with those of Chen Yun’s [17] survey in 2016, which evaluated the QoL of 486 elderly patients with hip fracture. That study showed that the QoL of elderly patients with hip fractures was at a moderate to low level three months after surgery. Due to influencing factors (such as poor physical function, the coexistence of multiple diseases, pain, and long-term bed rest), elderly patients with bone trauma had a long recovery time, a low physiological function score, poor recovery of limb function, and a poor ability to take care of themselves. As time went on, the patients’ scores on all QoL dimensions increased in our study. This result is consistent with the survey on HRQoL of patients after total hip arthroplasty, which was conducted by Shi HY in 2009. That study showed a significant improvement in the SF-36 score of the patients 6 months after discharge [18].

Our research also showed that the patients' total QoL score decreased 1 month after discharge and then gradually increased. This may be related to the patients’ health conditions at discharge, the type of care after discharge, family support and other factors. The previous relevant research of our team showed that when surgical patients were discharged after surgery, their postoperative recovery quality scores were lower than those before surgery. Those patients still have a high requirement for care [19]. Bone trauma patients often need professional rehabilitation treatment after discharge. However, most patients in this study chose to undergo rehabilitation at home. Therefore, they lack professional rehabilitation guidance. It has a great impact on the recovery of physiological functions, which results in low QoL scores.

Many factors affect the QoL scores of elderly patients with bone trauma. The main factors include the location of the trauma and the destination after discharge. Regarding the location of bone trauma, the patients with upper limb fractures had the highest QoL scores, while those with pelvic fractures had the lowest scores. Patients with pelvic fractures often require more complicated treatment, longer recovery time, and need to remain in bed for a long time. This will have a great impact on their QoL [20]. A study conducted by Tarride J E [10] in Canada in 2016 also found that the HRQoL of elderly patients with pelvic fractures did not return to the level before fracture, even at 36 months after the injury. Therefore, continuous care and long-term rehabilitation treatment after discharge are essential for elderly patients with pelvic fractures.

Regarding the destination after discharge, the QoL of patients who continue rehabilitation at home after discharge is higher, while at subordinate hospitals or nursing homes, it is lower. Patients transferred to subordinate hospitals after discharge often suffer more serious injuries, have worse body functions, and need long-term rehabilitation treatment. Therefore, these patients have lower QoL. Due to China's medical system and care methods, as well as the lack of professional rehabilitation institutions and the imperfect medical insurance reimbursement mechanism, most patients choose to continue treatment at home after discharge [21]. Early geriatric services, specialized trauma care, and geriatric trauma consultation services can help reduce the chances of staying in long-term care facilities after discharge [22, 23]. Therefore, when elderly patients with bone trauma return home, medical staff in hospitals and communities should provide professional care, rehabilitation guidance, and social support resources. It can significantly improve the QoL of patients.

In addition, surgical status is also one of the factors affecting the long-term (6 months after injury) QoL of elderly patients with bone trauma. Elderly patients who choose to undergo surgery after injury have higher QoL scores. Some scholars believe that the timing of surgery is not related to postoperative complications or mortality [24]. However, some studies have shown that prompt surgery after injury can quickly correct fracture displacement, rebuild joint function, improve the fracture healing rate, effectively relieve pain, shorten bed rest time, and improve QoL [25]. In this study, 80% of the patients underwent surgical treatment, and no deaths occurred. A study in the UK in 2006 showed that in elderly patients with hip fractures, if surgery is delayed for more than 4 days, the mortality rate will significantly increase [26]. Another study in the United States in 2016 suggested that markers of resuscitation (such as pH and base excess) may dictate the appropriate timing of surgery [27]. However, whether surgery should be performed for elderly patients with bone trauma still requires comprehensive evaluation of the patient's condition and health status, the medical team's treatment capabilities and other factors.

Due to time and researcher limitations, in this study, only a six-month follow-up survey of elderly patients with bone trauma was conducted, and only 100 hospitalized patients were included. A larger sample size and longer investigation period may lead to more diverse results on the long-term QoL outcomes of elderly bone trauma patients. Meanwhile, because of admitted diseases, the bone trauma included in this research was mainly musculoskeletal injuries of the pelvis and extremities. There was no indication of thoracic or cervical spine trauma. The QoL of patients may vary depending on the location of the bone trauma. In the future, we would like to carry out relevant research in multiple hospitals, including more elderly patients with bone trauma and focusing on elderly patients with cervical and thoracic trauma. We would like to conduct a longer follow-up survey. Perhaps we can obtain more interesting results.

## Conclusion

This study aimed to explore long-term QoL and its influencing factors in elderly patients with bone trauma. We found that the QoL of elderly patients with bone trauma was low at discharge, and the main factors influencing QoL were the location of bone trauma, the destination after discharge and surgical status. This study has certain limitations, such as insufficient follow-up time and small sample size, which may affect the analysis of long-term QoL for those patients. Our research still reveals the changes in the QoL of patients after bone trauma and the important influencing factors. At the same time, our findings indicate that hospital staff and community and nursing home staff should provide continuous care and rehabilitation services for elderly patients with bone trauma after discharge. Follow-up services should continue for at least six months for patients who undergo home rehabilitation after discharge. Simultaneously, informing their rehabilitation status and providing guidance, comprehensive treatment, and rehabilitation services can improve their QoL. Meanwhile, longer follow-up periods, larger sample sizes, and more comprehensive bone trauma locations can be embraced in future studies.

## Data Availability

The datasets used or analysed during the current study are available from the corresponding author upon reasonable request.

## Abbreviations

QoL: Quality of life
EQ-5D: EuroQol Five Dimensions Questionnaire
HRQoL: Health-related quality of life
PF: Physiological function
RP: Role-physical
BP: Bodily pain
GH: General health
VT: Vitality
SF: Social functioning
RE: Role-emotional
MH: Mental health

## References

[1] Go KT, Cheng JY, Seah X, Goh MH, Teo LT, Cole E (2019). The Changing Epidemiology of Serious Trauma in the Elderly Population: An Increasing Concern of a Tertiary Hospital in Singapore. Ann Acad Med Singapore..

[2] Khurrum M, Chehab M, Ditillo M, Richards J, Joseph B (2021). Trends in geriatric ground-level falls: report from the national trauma data bank. J Surg Res.

[3] Zhou Y (2019). The value of postoperative rehabilitation nursing and functional exercise in patients with bone trauma. Electron J Pract Clin Nurs Sci.

[4] Zhao F, Tang B, Liu X, Zhang Z, Zhang L (2022). Validating the agreement between the geriatric trauma frailty index and four published frailty scores in the Chinese geriatric trauma population. BMC Geriatr.

[5] Maxwell A, Patel B, Suarez-Rodriguez L, Miller RS (2019). Frailty and prognostication in geriatric surgery and trauma. Clin Geriatr Med.

[6] Jarman H, Crouch R, Halter M, George P, Cole E (2022). Provision of acute care pathways for older major trauma patients in the UK. BMC Geriatr.

[7] Lustenberger T, Talving P, Schnuriger B, Eberle BM, Keel MJ (2012). Impact of advanced age on outcomes following damage control interventions for trauma. World J Surg.

[8] Gioffrè-Florio M, Murabito LM, Visalli C, Pergolizzi FP, Famà F (2018). Trauma in elderly patients: a study of prevalence, comorbidities and gender differences. G Chir..

[9] Jun L, Cuilin K (2019). Application of plate and screw internal fixation in the treatment of traumatic fracture of extremitie. China Modern Med..

[10] Tarride JE, Burke N, Leslie WD, Morin SN, Adachi JD, Papaioannou A, et al. Loss of health related quality of life following low-trauma fractures in the elderly. Bmc Geriatrics. 2016;16(1). 10.1186/s12877-016-0259-5. 10.1186/s12877-016-0259-5 PMC4837505 27093957

[11] Qixiang Z, Xiaofeng X, Yongmei C, Ting J, Siying W, Heya J (2022). Applicability of three quality of life scales in elderly patients after bone trauma surgery. J Trauma Surg.

[12] Krantz E, Wide U, Trimpou P, Bryman I, Landin-Wilhelmsen K (2019). Comparison between different instruments for measuring health-related quality of life in a population sample, the who monica project, gothenburg, sweden: an observational, cross-sectional study. BMJ Open.

[13] Wang SJ, Wang YH, Huang LC (2021). Liquid combination of hyaluronan, glucosamine, and chondroitin as a dietary supplement for knee osteoarthritis patients with moderate knee pain: A randomized controlled study. Medicine (Baltimore).

[14] Christopher Colwell. Geriatric trauma: Initial evaluation and management. In: UpToDate. 2023. https://www.uptodate.com/contents/geriatric-trauma-initial-evaluation-and-management?search=Geriatric%20trauma:%20Initial%20evaluation%20and%20management&source=Out%20of%20date%20-%20zhHans&selectedTitle=1~150#disclaimerContent. Accessed 10 May 2023.

[15] Clare D, Zink KL (2021). Geriatric Trauma. Emerg Med Clin North Am.

[16] Stonko DP, Etchill EW, Giuliano KA, DiBrito SR, Eisenson D, Heinrichs T (2021). Failure to Rescue in Geriatric Trauma: The Impact of Any Complication Increases with Age and Injury Severity in Elderly Trauma Patients. Am Surg.

[17] Yun C (2017). The Status of Post-Traumatic Growth and Post-Traumatic Stress Disorder in the Elderly Patients with Hip Fracture and Its Influence on Quality of Life.

[18] Shi HY, Khan M, Culbertson R, Chang JK, Wang JW, Chiu HC (2009). Health-related quality of life after total hip replacement: a Taiwan study. Int Orthop.

[19] Xu X, An J, Zhang Y, Li L, Yongmei C, Rengrong G (2022). Investigation of the Quality of Recovery of Surgical Patients Based on the Chinese Version of the Quality of Recovery-15 Survey, a Cross-Sectional Study. J Perianesth Nurs.

[20] Kavak N, Duman E, Tikman M, Yaman AS (2021). Evaluation of epidemiological characteristics of pelvic fractures. Turk J Clin Lab.

[21] Yun Z (2021). Study on the adaptation theory of medical service system and medical insurance payment method in China. Chin Health Serv Manage.

[22] Lampron J, Khoury L, Moors J, Nemnom MJ, Figueira S, Podinic I, et al. Impact of a geriatric consultation service on outcomes in older trauma patients: a before–after study. Eur J Traum Emerg Surg. 2021:1–7. 10.1007/s00068-021-01724-x. 10.1007/s00068-021-01724-x 34146122

[23] Lenartowicz M, Parkovnick M, McFarlan A, Haas B, Straus SE, Nathens AB (2012). An Evaluation of a Proactive Geriatric Trauma Consultation Service. Ann Surg.

[24] Kanthasamy S, To K, Webb JI, Elbashir M, Parker MJ (2022). Timing of surgery for internal fixation of intracapsular hip fractures and complications at 1 year; a 32 year clinical study of 2,366 patients at a single center. Injury.

[25] Seckel T, Mahoney K, Hewitt C, Liu H, Ang D. Outcomes after definitive surgery for nonagenarians with isolated hip fractures within 24 hours of admission. Am Surg. 2022;0(0). 10.1177/00031348211067994. 10.1177/00031348211067994 35282709

[26] Moran CG, Wenn RT, Sikand M, Taylor AM (2006). Early Mortality After Hip Fracture: Is Delay Before Surgery Important?. J Bone Joint Surg.

[27] Reich MS, Dolenc AJ, Moore TA, Vallier HA (2016). Is Early Appropriate Care of axial and femoral fractures appropriate in multiply-injured elderly trauma patients?. J Orthop Surg Res..

